# Molecular pathogenesis of *Cryptosporidium* and advancements in therapeutic interventions

**DOI:** 10.1051/parasite/2025001

**Published:** 2025-02-04

**Authors:** Yilong Lu, Xiaoning Zhang, Zhiyu Guan, Rui Ji, Fujun Peng, Chunzhen Zhao, Wei Gao, Feng Gao

**Affiliations:** 1 College of Basic Medical Sciences, Shandong Second Medical University Weifang China; 2 College of Pharmacy, Shandong Second Medical University Weifang China; 3 College of Traditional Chinese Medicine, Shandong Second Medical University Weifang China; 4 College of Clinical Medicine, Shandong Second Medical University Weifang China

**Keywords:** *Cryptosporidium*, Cryptosporidiosis, Signaling pathways, Enzymatic targets, Drug therapy

## Abstract

Cryptosporidiosis, caused by a *Cryptosporidium* infection, is a serious gastrointestinal disease commonly leading to diarrhea in humans. This disease poses a particular threat to infants, young children, and those with weakened immune systems. The treatment of cryptosporidiosis is challenging due to the current lack of an effective treatment or vaccine. Ongoing research is focused on understanding the molecular pathogenesis of *Cryptosporidium* and developing pharmacological treatments. In this review, we examine the signaling pathways activated by *Cryptosporidium* infection within the host and their role in protecting host epithelial cells. Additionally, we also review the research progress of chemotherapeutic targets against cryptosporidia-specific enzymes and anti*-Cryptosporidium* drugs (including Chinese and Western medicinal drugs), aiming at the development of more effective treatments for cryptosporidiosis.

## Abstract (fr)

La cryptosporidiose, causée par une infection à *Cryptosporidium*, est une maladie gastro-intestinale grave entraînant généralement une diarrhée chez l’homme. Cette maladie constitue une menace particulière pour les nourrissons, les jeunes enfants et les personnes dont le système immunitaire est affaibli. Le traitement de la cryptosporidiose est difficile en raison de l’absence actuelle de traitement ou de vaccin efficace. Les recherches en cours se concentrent sur la compréhension de la pathogénèse moléculaire de *Cryptosporidium* et le développement de traitements pharmacologiques. Dans cette revue, nous examinons les voies de signalisation activées par l’infection à *Cryptosporidium* au sein de l’hôte et leur rôle dans la protection des cellules épithéliales de l’hôte. De plus, nous passons également en revue les progrès de la recherche sur les cibles chimiothérapeutiques contre les enzymes spécifiques de *Cryptosporidium* et les médicaments anti-*Cryptosporidium* (y compris les médicaments chinois et occidentaux), en visant le développement de traitements plus efficaces contre la cryptosporidiose.

## Introduction

*Cryptosporidium*, one of the most widely distributed intestinal parasites globally [166], was first defined as a human pathogen in 1976 [145]. Human infections with *Cryptosporidium* are predominantly caused by *C. hominis* and *C. parvum* [62]. Belonging to the Coccidia class within the phylum Apicomplexa, *Cryptosporidium* possesses distinct features that distinguish it from other members of the Coccidia group [83]. Its intracellular and extracytoplasmic localization is a key characteristic, along with the formation of unique “feeding” structures. In addition, *Cryptosporidium* produces two morphologically distinct types of oocysts, *i.e.*, thick-walled and thin-walled, which differ markedly from the oocysts of other Coccidia by lacking structures like sporocysts or micropyles. Despite the common use of chlorine as a disinfectant in water to eliminate numerous microorganisms, *Cryptosporidium* oocysts exhibit resistance to chlorine and various environmental stresses, such as extreme temperature changes [77]. This resilience enables the oocysts to remain infectious outside of a host for long periods.

*Cryptosporidium* infection can lead to damage to intestinal epithelial cells in both humans and other mammals. This damage disrupts the tight junctions of the intestinal epithelium, affecting intestinal barrier function. It also hinders the development of villi and reduces the absorptive capacity of the intestinal surface, leading to malnutrition, dehydration, and diarrhea [105]. The diarrheal symptoms associated with cryptosporidiosis pose a major threat to human health [103]. Malnourished children are particularly susceptible to *Cryptosporidium* infection, which can lead to recurrent or persistent infections and even death [40, 108, 127]. In adults, cryptosporidiosis commonly occurs in immunocompromised individuals, especially people with AIDS and organ transplant recipients [49].

*Cryptosporidium* infection is a widespread global disease, with a global pooled prevalence of 7.6% [51]. The World Health Organization (WHO) classified the disease as one of the neglected diseases in 2004 [162] and identified it as one of the six major diarrheal diseases [196]. Cryptosporidiosis is more commonly found in developing countries compared to developed nations [17]. It causes more than 50 million episodes of diarrhea and 48,000 deaths annually among children under 5 years of age in developing countries, especially in sub-Saharan Africa [66, 101]. The infection is also prevalent among immunocompromised populations in low- and middle-income countries, notably among HIV-positive individuals [9, 121, 139, 172, 178]. In Africa, the prevalence of cryptosporidiosis among people with HIV ranges from 5.6% to 25.7%; in Asia, from 3.7% to 45.0%; in South America, from 5.6% and 41.6%; and in Europe, from 2.6% to 15.1% [178].

*Cryptosporidium* infection is mainly transmitted through the fecal-oral route and can occur due to contact with animals, feces, or contaminated water or food [160]. Respiratory secretions and aerosolised droplets from coughing can also serve as means of transmissions, leading to pulmonary infections [67]. The pathogen poses a significant threat to the water industry [38], with nearly 35% of reported cryptosporidiosis outbreaks in the United States between 2009 and 2017 linked to exposure to treated recreational water sources, such as swimming pools and water playgrounds. This exposure was associated with nearly 57% of the reported cases during this period [68]. A recent report from Kenya indicated an outbreak of diarrhea caused by *Cryptosporidium hominis* infection likely stemming from contact with contaminated waters during swimming [170]. Contact with infected cattle and people in childcare settings can also spark outbreaks [68]. Additionally, *Cryptosporidium* is recognized as an important foodborne pathogen, contributing to over 40 recorded foodborne outbreaks and more than 8 million cases of foodborne illness annually [200]. These factors are considered high-risk factors for *Cryptosporidium* infection (Table 1).

**Table 1:** Risk factors for *Cryptosporidium* infection.

Common infection risk factors	References
Risk factors for *Cryptosporidium* infection	
Entertainment water exposure	[27]
Eating contaminated food	[30]
Animal contact	[28]
Drinking contaminated groundwater	[77]
Contact with infected persons	[197]
Contact with infected individuals in childcare environments	[68]
Travelling abroad	[64]
Contact with farm or domestic animals	[167]
Overcrowding and shared toilets	[107]
Risk factors for *Cryptosporidium* infection in HIV/AIDS	
CD4 cell count <200 cells/mL	[111]
Male sex	[36]
Anal sex	[82]
Multiple partners	[31, 82]
Intravenous drug use	[20]

The objective of this review was to comprehensively analyze the molecular mechanisms underlying *Cryptosporidium* infection. This includes elucidating the signaling pathways activated by *Cryptosporidium* and their role in host defense mechanisms against the infection. Furthermore, we aimed to discuss potential chemotherapeutic targets and recent advancements in drug development for *Cryptosporidium*. Uncovering the unique metabolic pathways and survival strategies of *Cryptosporidium* has led to the identification of targets for specific enzymes, resulting in significant strides in drug development against this parasite. In addition, we offer an overview of existing research on drugs targeting *Cryptosporidium* infections, ranging from Chinese herbal remedies to clinical therapeutics. Through this review, we aspire to provide scientific insight and theoretical support to facilitate the development of more effective treatments and medications for cryptosporidiosis.

## Molecular pathogenesis of *Cryptosporidium*

To date, more than 200 drugs have been screened for cryptosporidiosis, but an optimal therapeutic intervention remains elusive. While several vaccine candidates have demonstrated limited protective effects through targeting specific genes [40], their efficacy falls short of therapeutic requirements. This situation fully demonstrates the urgent need for new therapeutic strategies to address this pressing public health challenge. To overcome this challenge, future research on *Cryptosporidium* should focus more on its molecular mechanisms, especially the signaling pathways implicated in the process of *Cryptosporidium* infection. Understanding how these signaling pathways are regulated and how they contribute to the activation of host defense mechanisms is crucial for the control and prevention of *Cryptosporidium* infection. Delving deeper into this field will establish a solid theoretical basis for the creation of innovative therapeutic strategies.

### The NF-κB pathway plays a role in regulating host epithelial cell RNA during *Cryptosporidium* infection

Activation of the NF-κB pathway is a critical response of epithelial cells to pathogen infections. NF-κB is an important group of nuclear transcription factors consisting of five members, namely p50, p52, p65 (Rel A), c-Rel, and Rel B [69]. In its inactivated state, NF-κB is bound to the inhibitory factor IκB and remains inactive in the cytoplasm [78]. Various stimuli, such as bacterial, viral, and protozoan parasitic infections, can trigger the activation of NF-κB [37]. Upon activation, NF-κB translocates to the nucleus, where the transcription factors specifically bind to the κB site in the promoter region of target genes. This binding initiates gene transcription, regulating the expression of a variety of host genes [211, 212].

**Figure 1:**
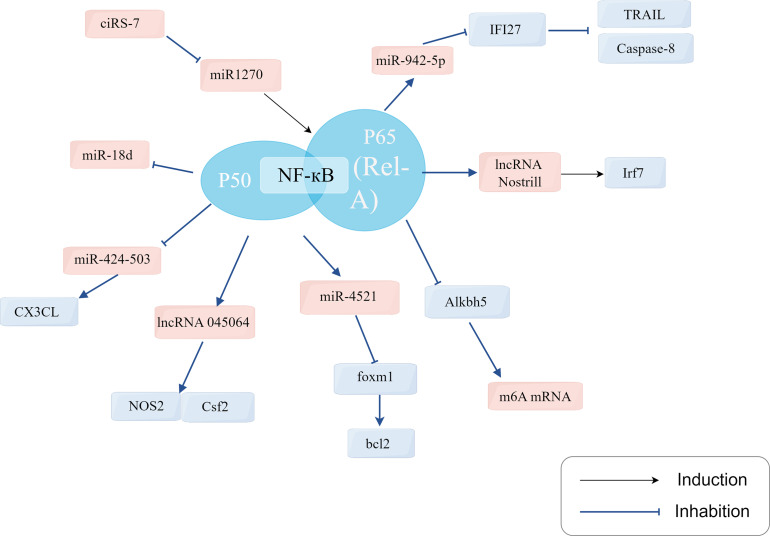
Schematic diagram of the NF-κB pathway involved in the regulation of host epithelial cell RNA during *Cryptosporidium* infection.

Host infection with *Cryptosporidium* activates the NF-κB signaling pathway. Ongoing research is uncovering the mechanisms through which NF-κB signaling regulates the role of epithelial cell RNA in defense against *Cryptosporidium* infection. This includes the regulation of mRNA, miRNA, lncRNA, and circRNA (Fig. 1). A recent study has identified a new role for NF-kB signaling in controlling CX3CL1 expression during *Cryptosporidium parvum* infection. The expression of CX3CL1 is increased in biliary epithelial cells during *Cryptosporidium parvum* infection, and this induction may be associated with the downregulation of miR-424 and miR-503. The downregulation of miR-424 and miR-503 is synergistically regulated by the NF-kB signaling pathway, leading to the increased expression of CX3CL1. The release of CX3CL1 contributes to the enhancement of defense against *Cryptosporidium parvum* by attracting immune cells to the mucosa [210].

The NF-κB signaling pathway is involved in the regulation of circRNAs in *Cryptosporidium*-infected host cells, offering a new perspective on the regulatory mechanisms of *Cryptosporidium* infection. In a study, it was observed that ciRS-7 was significantly upregulated in HCT-8 cells after *Cryptosporidium parvum* infection. RelA, a subunit of the NF-κB signaling pathway, plays a role in inhibiting apoptosis in epithelial cells, which can benefit parasite reproduction [41]. RelA is a direct target of miR1270, and ciRS-7 has the ability to affect the NF-κB pathway, promoting *Cryptosporidium parvum* reproduction by modulating the miR-1270/RelA axis [195].

Elevated levels of TLR4 and NF-κB have been observed in *Cryptosporidium parvum*-infected cholangiocytes, confirming the activation of the TLR/NF-κB signaling pathway [42]. Studies have revealed that the TLR2/TLR4-NF-κB signaling pathway is involved in the expression of miR-942-5p [179, 208]. Changes in miR-942-5p expression after *Cryptosporidium parvum* infection play a key role in the immune response of host epithelial cells. Activation of this pathway by *Cryptosporidium parvum* leads to the upregulation of miR-942-5p, and inhibition of TLR2 and TLR4 results in the significant downregulation of miR-942-5p. Additionally, miR-942-5p, which targets the IFI27 gene, has been shown to attenuate apoptosis in HCT-8 cells early in *Cryptosporidium parvum* infection, influencing the burden of *Cryptosporidium parvum* infection [188]. Additionally, *Cryptosporidium parvum* can mediate the expression of miR-181d in the TLR2/TLR4-NF-κB signaling pathway via the p50 subunit [61]. miR-181d expression is initially reduced during infection, while TLR2, TLR4, NF-κB, and myD88 in the pathway are upregulated. After inhibition of NF-κB and p50 subunit, miR-181d expression is increased. Recent findings suggest that the TLR/NF-κB signaling pathway contributes to the upregulation of miR-4521 expression during *Cryptosporidium parvum* infection. miR-4521 regulates BCL2-mediated apoptosis and targets FOXM1 to enhance *Cryptosporidium parvum* reproduction in HCT-8 cells [194]. This study provides a new theoretical framework for understanding the involvement of the NF-κB signaling pathway in the role of miRNA in *Cryptosporidium* infection.

In a study by Nicholas W. Mathy *et al*., it was found that *Cryptosporidium parvum* infection of the host intestinal epithelium leads to a significant increase in the expression of lncRNA Nostrill [135]. The study demonstrated that inhibiting the upregulation of Nostrill and the downregulation of the NF-kB response gene Cxcl2 using an NF-kB inhibitor was effective, highlighting the crucial role of NF-kB signaling. Furthermore, Nostrill was shown to promote the recruitment of NF-kB p65 to the Irf7 gene promoter, thereby promoting Irf7 expression and strengthening the defense of the small intestinal epithelium against *Cryptosporidium parvum* [135]. Another report examining lncRNA expression in intestinal epithelial cells after *Cryptosporidium parvum* infection revealed that lncRNA NR_045064, which is controlled by NF-κB signaling, was significantly upregulated. Functionally, the induction of NR_045064 was found to enhance the transcription of defense genes and inhibit *Cryptosporidium parvum* infection [114]. These findings suggest that the NF-κB pathway plays a role in regulating lncRNA-mediated defense in intestinal epithelial cells against *Cryptosporidium* infection, indicating a promising avenue for future research in anti-infection strategies.

The TLR/MyD88/NF-кB signaling pathway in intestinal epithelial cells has been identified as a critical component of defense against *Cryptosporidium parvum* infection [30, 41]. When *Cryptosporidium parvum* infects the intestinal epithelium, it triggers the activation of the NF-кB signaling pathway, leading to the downregulation of Alkbh5, which is responsible for elevated levels of mRNA m^6^A methylation [186]. m^6^A methylation plays an important role in the regulation of the immune response [74] and enhances the defense against *Cryptosporidium parvum* in epithelial cells. MyD88, a key gene upstream of the NF-кB pathway, is involved in this process, and the levels of Alkbh5 expression were not seen in MyD88 knockdown intestinal epithelial cells. Chromatin immunoprecipitation (ChIP) analysis revealed a high occupancy of the Alkbh5 gene by the NF-кB subunit p65, indicating that Alkbh5 induces m^6^A methylation under the influence of the activated NF-кB pathway [186].

Understanding whether the regulation involved in *Cryptosporidium*-induced NF-kB signaling benefits the host, the parasite, or both is crucial. Clarifying these dynamics could help to develop more effective strategies to combat *Cryptosporidium* infection.

### The PI3K/Akt signaling pathway is involved in autophagy regulation during *Cryptosporidium* infection

EGFR is a tyrosine kinase receptor located in the cell membrane that plays a role in the activation of various signaling systems, primarily through the MAPK pathway and the PI3K/Akt pathway [181]. Upon stimulation, cellular EGFR becomes phosphorylated and triggers the PI3K/Akt signaling pathway, which is essential for regulating basic cellular functions [84]. In the context of *Cryptosporidium parvum*, the parasite has been found to activate the EGFR-PI3K/Akt pathway to manipulate host cell autophagy [193]. Autophagy is a cellular mechanism used for defense against invading pathogens [23], but parasites can evade the host immune response by manipulating this process [156]. Studies have shown that when HCT-8 cells are infected with *Cryptosporidium parvum* for 2–8 h, there is an elevated expression of EGFR-P and Akt-P (phosphorylated forms of EGFR and Akt, respectively). Inhibition of EGFR results in a significant reduction in the phosphorylation levels of EGFR and Akt. Akt, a downstream effector of PI3K, is activated through PI3K-mediated phosphorylation [168]. Inhibition of PI3K leads to a reduction in Akt expression. Additionally, LC3 serves as a crucial marker in the autophagic process [131]. When EGFR and Akt are blocked, upregulation of LC3 expression enhances autophagy and inhibits parasite proliferation [193]. This suggests that *Cryptosporidium parvum* can counteract autophagy by manipulating the EGFR-PI3K/Akt pathway to maintain its survival within the host. Research has also shown that *Cryptosporidium parvum* induces cellular autophagy through the PI3K/Akt/mTOR signaling pathway. Transmission electron microscopy has revealed the presence of autophagosomes in *Cryptosporidium parvum*-infected cells, and a decrease in the phosphorylation levels of PI3K, Akt, and mTOR proteins was observed after 8 h. The use of rapamycin (Rapa), an mTOR inhibitor, was able to inhibit mTOR signaling [3]. In cells pretreated with Rapa, the proliferation of *Cryptosporidium parvum* was significantly inhibited at 12 h post-infection. This indicates that *Cryptosporidium parvum* induces autophagy via the PI3K/Akt/mTOR pathway in HCT-8 cells, and cellular autophagy inhibits the early development of *Cryptosporidium parvum* [116]. While research on the interaction between *Cryptosporidium* and the PI3K/Akt pathway is in its infancy, further expansion in this area may reveal novel therapeutic and preventive strategies for combatting *Cryptosporidium* infections.

### The TLR4/STAT1 signaling pathway is involved in *Cryptosporidium* infection

Toll-like receptors (TLRs) play a key role in immune activation and pathogen recognition [10, 24], with elevated expression of TLR4 observed during *Cryptosporidium parvum* infection [189]. Recent studies, including our own research, have revealed an upregulation of TLR4 expression in a mouse model of *Cryptosporidium parvum* infection [97]. The TLR4-mediated responses are essential for the clearance of *Cryptosporidium parvum* bile duct infections *in vivo*. The absence of this pattern recognition receptor can lead to altered inflammatory responses and worsen hepatobiliary pathology [146]. STAT1 serves as a key regulator of IFN-γ signaling. During *Cryptosporidium parvum* infection, the parasite inhibits STAT1 activity [44, 154]. Additionally, the expression of interferon regulatory factor-1 (IRF-1) is reduced post-infection, impacting the regulation of various innate and adaptive immune responses. At the cellular level, IFN-γ can bind to its receptor (IFN-γR) to initiate signaling cascades and activate STAT1 [180]. A recent study has shown that Chitosan can target the TLR4/STAT1 pathway. By downregulating TLR4 signaling and upregulating the STAT1 signaling pathway to induce IFN-γ signaling, Chitosan can attenuate *Cryptosporidium parvum* infection [151]. This research sheds light on potential therapeutic strategies that target the TLR4/STAT1 pathway to combat *Cryptosporidium* infections effectively.

## Enzymatic targets for drug interventions against *Cryptosporidium* infection

Current therapeutic approaches for cryptosporidiosis are predominantly limited to pharmacological interventions. Nitazoxanide (NTZ), while approved by the FDA for treating human cryptosporidiosis, shows limited efficacy in immunocompromised and HIV-infected patients, necessitating the development of new therapeutic strategies. *Cryptosporidium*-specific enzymes, which play key roles in the parasite’s unique metabolic pathways and survival mechanisms, represent promising therapeutic targets. Our research focuses on these enzymatic targets (Table 2) with the aim of developing more effective treatments. However, the presence of homologous enzymes in mammalian cells presents a significant challenge, as inhibitors targeting these enzymes affect both parasite and host cellular functions, potentially triggering adverse effects. Therefore, drug development efforts must prioritize strategies that selectively target parasite-specific enzymes. To advance this objective, we will evaluate potential drugs against these targets through *in vitro* and *in vivo* experiments to provide a scientific basis for the development of novel therapeutic drugs for cryptosporidiosis.

**Table 2:** Enzyme targets and specific compounds targeted towards *Cryptosporidium*.

Related enzymatic targets	Targeted compounds	References
Lactate dehydrogenases (LDH)	NSC10447 and NSC158011, Gossypol and FX11	[115, 201]
Calcium-dependent protein kinas 1 (CDPK1)	BKI-1517, BKI-1553	[34, 163]
	BKI-1534, BKI-1649, BKI-1294, BKI-1369	[91]
	Other BKI analogues scaffolded with 5-aminopyrazole-4-carboxamide and pyrrolopyrimidine	[88, 90]
Plasmodium lipid kinase (PI(4)K)	KDU731	[130]
Nucleoside diphosphate kinase (NDK)	Ellagic acid	[32]
Inosine monophosphate dehydrogenase (IMPDH)	P131	[147]
Thymidylate-synthetase Dihydrofolate Reductase (TS-DHFR)	Modified pyrimidine derivatives	[110]
tRNA synthetases	BRD7929	[177]
	Compound 2093	[29]
	Compound 5	[22]
Subtilisin-like serine protease 1(SUB1)	Peptidic boronic acids	[117]
Cysteine Protease (Cryptopains)	K11777	[143]
Long-chain fatty acyl-coenzyme A synthetase (LC-ACS)	Triacsin C, R134	[39, 72]

### Lactate dehydrogenases (LDH)

The glycolytic pathway plays a key role in ATP production, with the lactate dehydrogenase (LDH) enzyme being pivotal in catalyzing the conversion of pyruvate to lactate and the oxidation of nicotinamide adenine dinucleotide (NADH to NAD+). The regenerative cycle of NAD+ within glycolysis is essential for generating ATP, a vital energy source for cell survival and development [63, 94, 134]. Parasitic mitochondria, including those of *Cryptosporidium parvum*, lack functional oxidative phosphorylation and a complete tricarboxylic acid (TCA) cycle. Consequently, these parasites rely on anaerobic respiration for their energy needs, with LDH playing a crucial role in meeting these requirements [100]. Thus, inhibiting LDH activity may potentially result in the death of *Cryptosporidium*. Lactate dehydrogenase inhibitors such as gossypol and FX11 demonstrate inhibitory activity against *Cryptosporidium parvum* lactate dehydrogenase (CpLDH), albeit without specificity [201]. Thus, inhibitors targeting CpLDH may also affect LDH activity in mammalian cells. While CpLDH shares structural similarities with human LDH, detailed structural analysis reveals distinct differences in both the active site and cofactor binding regions. These variations result in CpLDH exhibiting lower affinity for NAD+/NADH and may affect inhibitor selectivity [45]. Studies have identified NSC158011 and NSC10477 as selective CpLDH inhibitors that effectively suppress the growth and replication of *Cryptosporidium parvum* both *in vitro* and *in vivo*, demonstrating inhibitory effects at concentrations below the threshold of mammalian cell toxicity [115]. Given these findings, a comprehensive evaluation of this inhibitor’s potential effects on humans remains crucial in the development of CpLDH-targeted inhibitors.

### Calcium-dependent protein kinas 1 (CDPK1)

Similar to other eukaryotic cells, protein kinases play a crucial role in the biology of protozoan parasites such as *Cryptosporidium* [140], making them potential targets for anti-cryptosporidial drug development. Apicomplexan CDPKs are essential enzymes involved in parasite gliding motility, invasion, and exit [173]. However, the catalytic sites of these CDPKs contain a glycine instead of the bulkier gatekeeper residues typically found in mammalian CDPKs. Bumped kinase inhibitors (BKI) target parasitic CDPKs, and a series of inhibitor compounds have been developed [35]. Previous reports have shown that BKI-1517 and BKI-1553 exhibit anti-cryptosporidial activity in both *in vivo* and *in vitro* assays [34, 163]. Further investigations by Hulverson et al. on BKI-1294, BKI-1369, BKI-1534, and BKI-1649 revealed that all these compounds reduce *Cryptosporidium parvum* infections [91]. BKI-1294 reduces oocyst shedding but does not effectively address diarrhea and dehydration in calves [113]. BKI-1369 shows promising anti-cryptosporidiosis effects in the neonatal calf and piglet models infected with *Cryptosporidium parvum* [89, 112], positioning it as a potential candidate for disease treatment in animal models. Although these BKIs demonstrate potent inhibitory activity against the human ether-à-go-go-Related Gene (hERG), which is associated with human QT syndrome and cardiotoxicity, caution is advised in their use for human cryptosporidiosis treatment [173]. In addition, other BKI analogues structured with 5-aminopyrazole-4-carboxamide and pyrrolopyrimidines, have shown effectiveness in IFN-γ KO mouse models of *C. parvum* infection. However, these compounds exhibit certain pharmacological properties that preclude their immediate application in human treatments [88, 90]. Nevertheless, inhibitors targeting CpCDPK1 present compelling evidence for their therapeutic potential, establishing a promising direction for clinical research in cryptosporidiosis treatment.

### Plasmodium lipid kinase (PI(4)K)

Phosphatidylinositol-4-oh kinase (PI(4)K) is an enzyme found in the apicomplexan protozoa *Plasmodium*, where phosphorylated lipid molecules play crucial roles in intracellular signaling and transport [137]. Researchers have targeted the *Cryptosporidium* lipid kinase PI(4)K and, through screening efforts, have optimized a potential inhibitory compound known as the pyrazolopyridine derivative KDU731. KDU731 exhibits selective inhibition of this enzyme, effectively reducing oocyst shedding, diarrhea duration, and dehydration symptoms in experimentally infected calves, with no significant adverse effects observed [130]. Similarly, EDI048, another selective *Cryptosporidium* PI(4)K inhibitor, shows potent antiparasitic activity and demonstrates therapeutic efficacy when administered orally in animal models, successfully reducing oocyst shedding and ameliorating diarrhea symptoms [129]. Previous studies have established the effectiveness of both compounds in animal models, suggesting that successful progression through clinical trials could address the current therapeutic void in cryptosporidiosis treatment.

### Nucleoside diphosphate kinase (NDK)

*Cryptosporidium* nucleoside diphosphate kinase (NDK) is essential for the biosynthesis of nucleoside triphosphate and normal metabolism in *Cryptosporidium*, which can be used as a strategic target for therapeutic development. *In vitro* studies have demonstrated that NDK suppression significantly inhibited *Cryptosporidium* proliferation [32]. Additionally, the anti-*Cryptosporidium* activity of the NDK inhibitor ellagic acid (EA) was tested, with EA demonstrating a reduction in *Cryptosporidium* loads (EC_50_ = 15–30 μM) without showing human cytotoxicity [32]. While NDK is conserved across mammalian cells, preliminary observations demonstrate that EA exhibits substantially higher inhibitory activity against semi-purified *Cryptosporidium* NDK compared to its human homolog [32], establishing a basis for the development of selective *Cryptosporidium* NDK inhibitors. Further research by Castellanos-Gonzalez *et al*. introduced a single-stranded RNA (ssRNA)/Argonaute (Ago) complex to silence *Cryptosporidium parvum* NDK in both *in vivo* and *ex vivo* models. The study showed that ssRNA/Ago attenuates *in vitro Cryptosporidium parvum* infections and reduces *Cryptosporidium parvum* burden in infected mice, suggesting its potential as a treatment for cryptosporidiosis [33].

### Inosine monophosphate dehydrogenase (IMPDH)

Inosine monophosphate dehydrogenase (IMPDH) is an enzyme essential for catalyzing the conversion of inosine monophosphate (IMP) to xanthine monophosphate (XMP), a pivotal and rate-limiting step in guanine nucleotide biosynthesis [81]. Guanine nucleotides serve as precursors for glycosylation, RNA, DNA and tetrahydrobiopterin synthesis across various organisms [13, 14].Considering the significance of guanine nucleotides, inhibition of *Cryptosporidium* IMPDH represents a promising therapeutic strategy for cryptosporidiosis. The distinctive evolutionary origin of *Cryptosporidium* IMPDH, which is structurally divergent from its host counterpart, provides a molecular foundation for developing selective inhibitors that can effectively target the parasitic enzyme, while minimizing interference with host cellular functions [104]. P131, an inhibitor of *Cryptosporidium* IMPDH, has displayed potent anti-cryptosporidial activity in mice [70] and exhibits selective binding to the NAD site within *Cryptosporidium* IMPDH [147]. Moreover, a peptide-based inhibitor known as phylomer has shown efficacy in targeting *Cryptosporidium* IMPDH, with phylomers 8 and 24 demonstrating the inhibition of *Cryptosporidium parvum* proliferation in HCT-8 cells [96]. In a study by Sarwono *et al*., three irreversible IMPDH inhibitors (disulfiram, bronopol, and ebselen) were screened. Oral administration of disulfiram and bronopol in a severe combined immunodeficiency (SCID) mouse model of cryptosporidiosis was found to reduce oocyst excretion [161]. A follow-up study suggested the presence of a potentially novel enzyme in the parasite that might utilize a unique mechanism to synthesize XMP, potentially circumventing the need for IMPDH [149]. This discovery indicates that drug development targeting *Cryptosporidium* IMPDH must address dual considerations, namely the specificity relative to host IMPDH and the possibility of metabolic pathways that could circumvent IMPDH inhibition.

### Thymidylate-synthetase dihydrofolate reductase (TS-DHFR)

Studies on *Cryptosporidium* nucleotide metabolism have identified a variety of proteins crucial for the parasite’s life cycle. Among these, thymidylate synthase-dihydrofolate reductase (TS-DHFR), a bifunctional enzyme involved in the synthesis of monophosphate thymine and folate in *Cryptosporidium*, stands out. The enzyme exists as a homodimer, with each monomer containing a unique swap domain known as a “crossover helix”, which is not present in human DHFR [132]. This structural divergence makes TS-DHFR an attractive target for selective inhibitor development [71, 133, 199]. Modified pyrimidine derivatives have been evaluated as inhibitors of *Cryptosporidium hominis* (Ch) TS-DHFR, demonstrating an EC_50_ of 1–5 μM in cell cultures. These inhibitors significantly impeded parasite proliferation [110]. Additionally, Ruiz et al. used virtual screening and structure-guided modeling to identify compounds targeting ChTS-DHFR, leading to the discovery of 15D17 as the first covalent inhibitor designed to target the non-catalytic pocket of ChTS-DHFR [159]. These studies emphasize the development of selective inhibitors targeting *Cryptosporidium*-specific enzymes, while minimizing interactions with host cell homologs, establishing novel therapeutic approaches for cryptosporidiosis treatment.

### tRNA synthetases

A tRNA synthetase is responsible for binding specific amino acid residues to selected tRNAs, a fundamental step in protein synthesis. Inhibitors targeting tRNA synthetases have been explored in the development of anti-cryptosporidial drugs, such as those focused on prolyl-tRNA synthetase [95] and leucyl-tRNA synthetase [148]. Vinayak *et al*. found that BRD7929, a bicyclic azopyridine compound targeting *Cryptosporidium* phenylalanyl-tRNA synthetase, showed significant anti-cryptosporidial effects in both *in vitro* and *in vivo* experiments [177]. Another research study revealed that compound 2093, a *Cryptosporidium* methionyl-tRNA synthetase inhibitor, displayed potent antiparasitic activity against *Cryptosporidium*, while concurrently inhibiting human mitochondrial methionyl-tRNA synthetase. Although short-term treatment may be tolerated, further investigation is warranted to fully evaluate its therapeutic potential and safety profile [29]. Despite the promising results, challenges such as recurrent infections and emerging resistance have been observed, particularly in *Cryptosporidium parvum*-infected calves treated with compound 2093 [75]. Therefore, the development of new treatment modalities should be accompanied by a focus on combating resistance. Studies have reported that compound 5, a cladosporin derivative, is a potent inhibitor of *Cryptosporidium* Lysyl tRNA synthetase (KRS), and also acts against human KRS (HsKRS), but with stronger activity against *Cryptosporidium parvum* (Cp) KRS [22]. While the compound significantly reduced oocyst shedding in murine models, administration at higher doses (50 mg/kg orally) induced toxicity, probably due to inhibition of mammalian KRS, resulting in disruption of protein synthesis, cell metabolism, and physiological functions, with potential implications for organ dysfunction. Although tRNA synthetase represents a potential target for anticryptosporidial therapeutics, the development of inhibitors must carefully address selective targeting of parasitic enzymes, as insufficient specificity may result in host enzyme inhibition, leading to adverse effects and potential health complications.

### Subtilisin-like serine protease 1 (SUB1)

Egress, the process by which motile merozoites are released from infected cells, is a key step in the *Cryptosporidium* life cycle, facilitating the spread of infection within the host epithelium. Research in *Plasmodium falciparum* has highlighted the role of Subtilisin-like serine protease 1 (SUB1) in cleaving parasite vesicle membranes (PVM) and host cytosolic membranes, promoting infection spread [8, 25]. Additionally, the *Plasmodium falciparum* SUB1 inhibitory compound, peptide boronic acid, has shown the ability to inhibit parasite replication *in vitro* [117]. Drawing parallels from plasmodium research, it was hypothesized that *Cryptosporidium* SUB1 may play a similar role in egress, making it a potential target for inhibiting *Cryptosporidium* infection. Reports have demonstrated that blocking SUB1 significantly inhibits *Cryptosporidium parvum* infection in cell culture [60]. A recent study has specifically identified SUB1 as a key mediator of *Cryptosporidium parvum* egress from host cells [142]. Nava et al. conducted *Cryptosporidium parvum* SUB1 mRNA silencing (ΔSUB1), leading to a 95% reduction in schizonts released *in vitro* compared to wild-type strains. Additionally, a higher number of intracellular parasites were observed in ΔSUB1 cells than in the wild-type control, indicating that SUB1 silencing effectively blocked *Cryptosporidium* egress and infection spread [142]. These findings suggest that targeting *Cryptosporidium* SUB1 could be a significant avenue for future vaccine and drug development efforts to combat *Cryptosporidium* infection and transmission.

### Cysteine protease (cryptopains)

In *Cryptosporidium parvum*, 20 genes encoding clan CA (papain-like) cysteine proteases (CPs) have been reported, of which 5 genes encode “Cryptopains”, including 3 cathepsin L-like members and 2 cathepsin B-like members [6]. However, only cryptopain-1 has been biochemically analyzed and was found to be actively transcribed and expressed in sporozoites (the infective stage of the parasite) [141]. A cysteine protease inhibiting compound, N-methyl piperazine-Phe-homoPhe-vinylsulfone phenyl (K11777), has been identified as orally bioavailable with a tolerable safety and toxicity profile [2]. K11777 has been shown to inhibit *Cryptosporidium parvum* growth in a concentration-dependent manner in a variety of mammalian cell lines, including MDCK, HCT8, I407, and CaCo-2 [143]. The compound displays favorable selectivity, with minimal cytotoxicity observed in MDCK cells at concentrations up to 100 μM and negligible effects on human cells. *In vivo* efficacy studies revealed that K11777 effectively treats cryptosporidial infections in murine models, significantly improving infection parameters and preventing disease recurrence.

### Long-chain fatty acyl-coenzyme A synthetase (LC-ACS)

Long-chain fatty acid acyl-coenzyme A synthetase (LC-ACS), a key enzyme in fatty acid metabolism, represents a potential novel therapeutic target for cryptosporidiosis intervention. Adenosine monophosphate (AMP) binds to long-chain (LC) fatty acyl coenzyme A synthetase (ACSs), facilitating the thioesterification reaction that converts free fatty acids and coenzyme A into fatty acyl coenzyme A [16]. ACS not only catalyzes this important reaction, but also serves as a fatty acid transporter, enabling the activation of acyl coenzyme A thioesters from fatty acids, which are crucial intermediates in various pathways [182, 213]. In mammalian systems, different ACS isoforms regulate tissue-specific fatty acid metabolism and storage in hepatic, adipose, and muscular tissues. *Cryptosporidium parvum* genome encodes three distinct LC-ACS isoforms, each serving stage-specific functions during parasite development [73]. Research conducted by Guo *et al*. demonstrated that Triacsin C effectively inhibits *Cryptosporidium parvum* ACS activity. The compound exhibits significant antiparasitic effects *in vitro* and greatly reduces oocyst burden in IL-12 knockout mouse models without apparent toxicity [72]. While Triacsin C inhibits LC-ACS activity both in humans and animals, its selective impact on lipid metabolism pathways, specifically preserving phospholipid fatty acid recycling, provides a mechanistic explanation for its favorable toxicity profile in animal studies. Recent studies have identified R134 as a potential candidate for targeting *Cryptosporidium parvum* LC-ACS enzyme isoforms with improved efficacy and pharmacokinetic parameters [39]; however, further experimental validation is required to definitively establish its anticryptosporidial activity.

## Research on Chinese and Western medicinal drugs against *Cryptosporidium* infection

Controlling cryptosporidiosis poses a significant challenge in both veterinary and human medicine. Nitazoxanide, approved by the US FDA for the treatment of cryptosporidiosis [103], is effective primarily in immunocompetent patients [7]. In our comprehensive review, we analyzed previously published studies on drugs for combating *Cryptosporidium* infection, including Chinese herbal remedies and clinical therapeutics (Tables 3 and 4). Our investigation spans a wide range of experiments, including those conducted in animal models and human clinical trials. The evaluation criteria considered in the experiments comprise clinical improvement (such as reduction in diarrhea, changes in body weight, and enhancement of overall host health) and parasite clearance (involving the clearance of host fecal oocysts). These assessments are crucial in determining the efficacy of the treatment against cryptosporidiosis.

**Table 3:** Anti-*Cryptosporidium* efficacy of different western medications in human clinical and host models.

Therapeutic drug	Experimental paradigm	Research type	Reference	Types of treatment	Efficacy			
					Clinical cure		Parasitological cure	
					Degree of diarrhea	Change in weight	Infection intensity	Oocyst shedding
Nitazoxanide	AIDS patients	CT	[52]	Therapeutic	+	+	+	
	Non-immunodeficient patients	CT	[157]	Therapeutic	+	+	+	
	HIV-negative child patients	CT	[15]	Therapeutic	−	−	±	
	goat neonates	IVS	[176]	Prophylactic	NR	−	+	
Nitazoxanide-octaarginine	HCT-8 cells	IVE	[144]	Therapeutic	NR		+	
Nitazoxanide-secnidazole	Swiss albino mice	IVS	[126]	Therapeutic	NR		+	
Clofazimine-nitazoxanide	Swiss albino mice	IVS	[58]	Therapeutic	NR		+	
Clofazimine	HIV-infected patients	CT	[203]	Therapeutic	NR		−	
	Severely immunocompromised HIV patients	CT	[93]	Therapeutic	−	NR	−	
Paromomycin	Patients with AIDS	CT	[184]	Therapeutic	+	NR	+	
	AIDS patients (CD4 cell < or = 150/mm)	CT	[85]	Therapeutic	±	NR	±	
	BALB/c mice	IVS	[18]	Therapeutic	NR		+	
	C57BL/6 mice	IVS	[122]	Therapeutic	NR		+	
Paromomycin sulfate	(HCT-8 and Caco-2) cells	IVE	[128]	Therapeutic	NR		+	
Azithromycin	Immunocompromised children	CT	[12]	Therapeutic	+		+	
Mefloquine	Swiss albino mice	IVS	[57]	Therapeutic/Prophylactic	NR		+	

Note: “CT” indicates clinical trial; “IVS” indicates *in vivo* studies; “IVE” indicates *in vitro* experiments; “NR” denotes no reported; “+” denotes significant improvement; “−” denotes no significant improvement; “±” denotes moderate improvement.

**Table 4:** Anti-*Cryptosporidium* effects of various traditional Chinese medicines in farm animals and host models.

Therapeutic drug	Experimental model type	Types of infection	Reference	Types of treatment	Efficacy			
					Clinical cure		Parasitological cure	
					Degree of diarrhea	Change in weight	Infection intensity	Oocyst shedding
Matrine	MDCK cell/BALB/c mice	Experimental	[202]	Therapeutic	NR		+	
	Rabbit	Experimental	[125]	Therapeutic	NR		±	
Oxymatrine	BALB/c mice	Experimental	[98]	Therapeutic	NR		+	
Garlic	Swiss albino mice	Experimental	[65]	Therapeutic/Prophylactic	NR		+	
	BALB/c mice	Experimental	[59]	Therapeutic	NR	+	+	
	Swiss albino mice	Experimental	[54]	Therapeutic	NR		+	
Halofuginone	HCT-8 cells	Experimental	[164]	Therapeutic	NR		+	
	Goat neonates	Experimental	[150]	Prophylactic	+	NR	+	
	Neonatal calves	Natural	[175]	Prophylactic	+	−	+	
Pomegranate extract	Neonatal calves	Natural	[183]	Therapeutic	+		+	
	Albino mice	Experimental	[4]	Therapeutic	NR		+	
	Swiss Albino mice	Experimental	[56]	Therapeutic	NR		−	
Curcumin	BALB/c mice	Experimental	[19]	Therapeutic	NR		+	
	HCT-8 cells	Experimental	[155]	Therapeutic	NR		+	
	C57BL/6 neonatal mice	Experimental	[152]	Therapeutic	NR		+	

Note: “NR” indicates no reported; “+” indicates significant improvement; “−” indicates no significant improvement; and “±” indicates moderate improvement.

### Drugs in use for clinical treatment

#### Nitazoxanide

Currently, nitazoxanide (NTZ) stands as the only FDA-approved drug specifically designed for the treatment of cryptosporidiosis. NTZ is a thiazole antiparasitic drug known for its broad spectrum of antiparasitic activity [26, 209]. The drug primarily disrupts the function of pyruvate:ferredoxin oxidoreductase (PFOR) [165]. NTZ acts as an inhibitor of PFOR, disrupting anaerobic energy transfer reactions and inhibiting parasite growth [87]. The precise mechanism by which NTZ hampers *Cryptosporidium* infection remains incompletely understood, as these parasites possess a unique PFOR with a fused c-terminal cytochrome P450 structural domain [158]. Several studies have delved into NTZ clinical trials targeting cryptosporidiosis, yielding significant results [52, 157]. However, in immunocompromised individuals, the duration of diarrhea is prolonged, and parasite clearance is less effective [1, 15]. Nevertheless, significant alleviation of diarrheal symptoms has been reported in HIV-seronegative children treated with NTZ [15]. Improving the effectiveness of medications is a crucial focus. Research has demonstrated that octaarginine (R8) favors the uptake of NTZ. Treatment of *Cryptosporidium parvum*-infected human ileocecal carcinoma cells (HCT-8) with NTZ-R8 has shown enhanced inhibition of *Cryptosporidium parvum* growth compared to NTZ alone [144]. To further explore the anti-*Cryptosporidium* effects of NTZ, animal experiments were conducted using selected appropriate animal models and standard experimental methods to validate the efficacy of NTZ. Treatment of *Cryptosporidium* sp.-infected goats with NTZ has proven to effectively reduce oocyst shedding, demonstrating a therapeutic effect [176]. This efficacy was similarly observed in a mouse model of *Cryptosporidium* spp. infection [21, 53, 190]. Therapeutic benefits can be achieved through the administration of medications alone, but combinations of medications may enhance their effectiveness. Studies have shown that the combination of secnidazole (SEC) with NTZ [126], as well as the combined use of low-dose clofazimine (CFZ) and half-dose NTZ [58], resulted in significant reductions in disease compared to NTZ monotherapy. Due to the diverse mechanisms of action exhibited by different drugs, combining them can amplify their efficacy, while reducing the risk of adverse effects associated with a single drug. In addition, drug combinations can elevate the success rate of treatment, shorten the treatment period, and enhance overall treatment outcomes, providing substantial benefits for managing cryptosporidiosis.

#### Clofazimine

Clofazimine (CFZ) is a drug known for its anti-inflammatory properties [198] and has traditionally been used in the treatment of leprosy [43]. Recent studies have explored the potential of CFZ in the treatment of cryptosporidiosis. Experiments conducted with CFZ in a mouse model of *Cryptosporidium parvum* infection have shown promise in ameliorating symptoms associated with cryptosporidiosis [58, 123]. However, Zhang *et al*. reported no significant therapeutic effect of CFZ in a phase 2a clinical trial involving HIV-infected individuals with cryptosporidiosis [203]. This lack of efficacy may be due to the dose or concentration of CFZ not reaching therapeutic levels. Additionally, CFZ has not been found to be effective in treating cryptosporidiosis in immunocompromised HIV populations [93]. To enhance the effectiveness of CFZ in cryptosporidiosis treatment, the dosage of CFZ needs to be increased. As CFZ is insoluble, poorly absorbed and utilized, its oral bioavailability can be improved through SNEDDS (Self-NanoEmulsifying Drug Delivery System) formulations [191]. Furthermore, using CFZ nanoparticle formulations to treat cryptosporidiosis may be another viable approach. These strategies offer promising avenues for the future utilization of CFZ in cryptosporidiosis treatment.

#### Paromomycin

Paromomycin, an antibiotic classified under the aminoglycoside group, has been studied in published controlled trials for the treatment of cryptosporidiosis [92]. Like other aminoglycoside antibiotics, paromomycin hampers protein synthesis by specifically binding to the 30S ribosomal subunit and exhibits a broad spectrum of activity against bacteria and certain protozoa [119]. Animal model experiments have showed that paromomycin reduces oocyst shedding and alleviates host symptoms [18, 21, 122]. *In vitro* studies have further confirmed the anti-cryptosporidial properties of paromomycin [128, 185]. Prior research highlighted the effectiveness of paromomycin against cryptosporidiosis in people with AIDS [76]. While two randomized, placebo-controlled clinical trials involving individuals with HIV did not result in complete cures, they did demonstrate some degree of clinical improvement [85, 184]. In the management of pediatric patients with cryptosporidiosis and chronic diarrhea, paromomycin typically leads to improvement in certain clinical symptoms and parasitological markers, although it is often found to be less effective than nitazoxanide [92].

#### Other drugs

Azithromycin has demonstrated greater efficacy in the treatment of cryptosporidiosis in children compared to other medications. Studies suggest that azithromycin may exhibit therapeutic benefits in both immunocompetent and immunocompromised children, leading to rapid improvements in clinical symptoms and the clearance of parasites [12, 86, 174]. Additionally, other drugs such as mefloquine (MQ) have been studied for their potential therapeutic effects in cryptosporidiosis. In an experiment, the administration of a half-dose combination of mefloquine and NTZ resulted in significant therapeutic effects in *C. parvum*-infected mice. These effects included significant reductions in *Cryptosporidium* fecal oocyst shedding, improvements in pathological lesions in the ileum, and the normalization of levels of two cytokines, IFN-γ and IL-17 [57].

### Promising Chinese herbal medicines for the future

#### Matrine

Matrine is a potent alkaloid extracted from *Sophora flavescens* [120], with a wide range of biological activities, including antibacterial, antiviral, anti-inflammatory, and immunomodulatory effects [80, 118, 204, 205]. In an experimental model of *Cryptosporidium cuniculus* infection, matrine has shown potential therapeutic benefits by reducing the number of oocysts shed in the host’s feces [125]. Following *Cryptosporidium parvum* infection of host intestinal epithelial cells, the expression of Toll-like receptors (TLRs) TLR2 and TLR4 is upregulated [192]. Matrine has been observed to inhibit the activation of these TLRs [206]. Research by Ji Rui *et al*. indicated that treatment with oxidized matrine led to a decrease in fecal excretion of oocysts in infected mice, significant repair of damaged intestinal villi structures, and a notable decrease in the relative expression of Toll-like receptors TLR2 and TLR4 [98]. Recent studies have shown that matrine can diminish inflammatory responses and oxidative stress by blocking the IL-6/STAT3 signaling pathway, thereby attenuating intestinal mucosal damage in rats with inflammatory bowel disease (IBD) [106]. Future investigations could explore whether matrine is able to attenuate *Cryptosporidium*-induced intestinal mucosal inflammation and damage through the IL-6/STAT3 signaling pathway.

#### Garlic

Garlic, an important species in the *Allium* genus, is noted for its unique medicinal properties. It contains a variety of active compounds such as phenolic compounds, saponins, and organosulfur compounds [169], known for their anti-inflammatory and antioxidant properties [55, 124]. Research has indicated that garlic holds medicinal potential in the treatment of intestinal parasitic infections [79, 102, 109]. In a study by Abouel-Nour *et al*., the immunological effects of *Allium sativum* on *Cryptosporidium parvum*-infected mice were investigated. The *Allium sativum* treatment group exhibited significantly elevated IL-5 levels and reduced IFN-γ levels, suggesting that garlic enhances the immune response and helps clear the infection [5]. Garlic is now recognized for its preventive and potentially therapeutic effects against cryptosporidiosis [54]. Gaafar demonstrated that preemptive oral administration of garlic two days before infection effectively reduced the number of *Cryptosporidium* oocysts in the feces and intestines of infected mice, partially normalizing intestinal structures [65]. Subsequent studies have reiterated the anti-cryptosporidial efficacy of garlic [59]. Elbahaie et al. observed the therapeutic effect of garlic by analyzing fecal samples from mice and performing examinations to count *Cryptosporidium* oocysts. They found that the garlic treatment group showed the highest percentage reduction in the number of oocysts, indicating the best therapeutic effect [54].

#### Halofuginone

Halofuginone is a synthetic derivative of febrifugine that was first isolated from *Dichroa febrifuga*, a traditional Chinese herb, and shows significant inhibitory activity against various protozoan parasites and cancer cells [48, 136]. It is utilized as an antiparasitic drug [207] and possesses anti-cryptosporidial properties. Research has demonstrated that, in animal models, administering moderate doses of halofuginone (100 μg/kg) from the day of infection up to 9 days post-infection as prophylaxis can reduce the severity of cryptosporidiosis, as manifested by decreased oocyst shedding, diarrhea, and mortality [150]. Additionally, the anti-cryptosporidial activity of halofuginone was verified in an *in vitro* assay, where the reduction in proliferation rate was observed at different time points (3, 15, 27 and 45 h post-infection). The study revealed a 68% decrease in proliferation rate at 15 hours compared to 3 h, with a more significant inhibitory effect observed at 27 and 45 h compared to 15 h [164]. In a study by Díaz *et al*., calf fecal samples were microscopically examined for *Cryptosporidium parvum* infection by antacid staining (Ziehl-Neelsen). The research revealed a significant reduction in the infection rate among halofuginone-treated calves, suggesting a preventive effect [50]. In addition, the study demonstrated that halofuginone served as an effective preventive agent against cryptosporidiosis in calves in a farm environment [47, 171, 175].

#### Pomegranate peel

The ripe peel of the pomegranate plant, known as pomegranate peel and belonging to the pomegranate family, is rich in antioxidant phytochemicals [11]. Pomegranate peel extract exhibits antioxidant, anti-inflammatory, antiviral, and antibacterial properties [187], along with anticoccidial activity [138]. Incorporating concentrated pomegranate extract (CPE) into the milk of newborn calves has been reported to provide resistance against *Cryptosporidium parvum* infection, alleviate diarrhea, and diminish intestinal symptoms associated with the infection [183]. In a study by Aboelsoued *et al*. [4], pomegranate peel was divided into white and red layers, both of which were treated with nanoparticles and transformed into a 3 g/mL solution of pomegranate peel in distilled water. This solution was administered to infected mice by gavage. The results indicated that both red and white pomegranate skin extracts were effective in reducing fecal oocyst excretion in infected mice, with the red skin extract showing superior efficacy. Additionally, the intestinal morphology of the mice treated with red skin extract was restored to a state resembling normal morphology. Pomegranate peel extract is rich in phenolic compounds with antioxidant properties [46]. The red skin extract, in particular, has high antioxidant activity and plays a significant role in improving biochemical markers and oxidative stress levels in *Cryptosporidium parvum*-infected mice, contributing to the restoration of their health status [4]. The therapeutic efficacy of pomegranate peel extract against *Cryptosporidium parvum* infection was further reaffirmed in a subsequent phase study [56]. To enhance the therapeutic efficacy of pomegranate peel extract, it is recommended to prioritize the red peel extract and combine it with nanoparticle technology to prepare the mixture. This formulation significantly improves absorption and utilization within the organism, thereby maximizing the therapeutic effect.

#### Curcumin

Curcumin, a natural polyphenolic compound extracted from turmeric root, exhibits various pharmacological effects such as antioxidant, anti-inflammatory [19, 99], and antiparasitic activities [153]. A study conducted by Sandamalie Ranasinghe *et al*. tested the anti-cryptosporidial properties of curcumin using human ileocecal adenocarcinoma cells (HCT-8). The results demonstrated that curcumin possessed significant anti-cryptosporidial effects with low cytotoxicity and dose-dependent efficacy [155]. Further verification of the therapeutic effects of curcumin was undertaken in a mouse model. The study revealed a significant reduction in the number of oocysts in the curcumin-treated group compared to the infected group, with a reduction rate of 74.70%. Additionally, there was an improvement in the structure of the ileocecal villi. Curcumin was also found to decrease the expression of IFN-γ and IL-18, while stimulating the antiparasitic activity of intestinal epithelial cells, thereby combating *Cryptosporidium parvum* infection [152]. Another independent experiment confirmed the therapeutic effects of curcumin in mice infected with *Cryptosporidium parvum* [19].

## Conclusion

Cryptosporidiosis, a severe gastrointestinal disease caused by *Cryptosporidium* infection, presents a significant threat to infants, young children, and immunocompromised individuals. The absence of an effective treatment or vaccine has posed a considerable challenge to public health. Current research is progressing towards understanding molecular mechanisms, emphasizing the exploration of activated signaling pathways post-infection and their regulatory functions in host defense against the parasite. It is necessary to further develop inhibitors targeting key components of these signaling pathways to disrupt essential signaling pathways crucial for the survival of *Cryptosporidium*, thereby impeding its ability to infect and replicate. Additionally, ongoing investigation into host defense signaling pathways is vital to enhance host cell resistance to infection. These research directions hold great promise and offer new strategies for the treatment of *Cryptosporidium* infections.

Nowadays, ongoing exploration is uncovering new chemotherapeutic targets, especially *Cryptosporidium*-specific enzymes that play crucial roles in the parasite’s metabolic pathways and survival mechanisms. To facilitate the development of novel compounds targeting these key enzyme classes, future research should prioritize the following aspects: (1) identification of potent and low-toxicity drug compounds through high-throughput screening and structure optimization; (2) exploration of the potential for multi-target drugs and the development of combination therapies that exert multiple effective actions; and (3) investigation into the resistance mechanisms of *Cryptosporidium* toward existing medications and the development of new drugs capable of overcoming resistance.

The discussion also extends to Chinese herbal medicine and the ongoing research progress in clinical treatment, determining the ideal combinations and dosages, and integrating Chinese herbal medicine with conventional drugs in clinical settings. Evaluation of the combined effects in model trials may offer fresh possibilities for clinical treatment. It is believed that through continuous research efforts and advancements in technology, novel breakthroughs in the treatment of cryptosporidiosis are on the horizon.
